# Imaging of central airway stenting with multidetector computed tomography

**DOI:** 10.1186/2049-6958-7-20

**Published:** 2012-07-26

**Authors:** Giovacchino Pedicelli

**Affiliations:** 1 Department of Diagnostic Imaging, S. Camillo-Forlanini Hospital, Rome, Italy

## 

When around 1846 the English dentist CT. Stent created a compound capable of alleviating the discomfort of his edentulous patients, he could not have imagined that over the years his name would have lost the capital letter and become synonymous with a "device" made of completely different material, destined to restore patency or prevent the tubular anatomical structures from collapsing.

The transition of the dental impression compound into a surgical tool is attributable to Johannes Fredericus Esser (1877 to 1946), a Dutch plastic surgeon who pioneered an innovative method of reconstructive surgery on soldiers with face wounds during the First World War. The material used was called "stents mold" having already lost the capital letter in the daily use. The first reference to a polyethylene tube “to act as a stent for the anastomosis” in experimental biliary reconstruction in dogs appeared in 1954. In 1966, for the first time the term "stent" [1] appeared in literature, with reference to a "rubber tube" inserted in the bile duct of a patient, consisting of a material that had lost any resemblance with that of Dr. Stent. In the same year the notion of stent found its way into cardiology: Weldon et al. [2] described "a prosthetic-stented aorthic homograft used for mitral valve replacement…", where the term stent was referred to "any kind of non-biological support used to give shape or form to biological tissue. " In 1986 the first self-expanding coronary endoprothesis would be implanted: that was the beginning of the triumphant era of vascular endoprothesis!

The “Montgomery T-tube” was introduced in 1965 dedicated to the treatment of subglottic stenosis subsequent tracheal surgery: it began with the perception of being able to overcome the numerous obstacles that can prevent the passage of air through the tracheo-bronchial axis by using innovative materials and technologies. The certainty arrived in 1990’s when Dr JF Dumon created a silicone "dedicated tracheobronchial stent". It provided specific characteristics: a flexible shape able to reproduce the natural tracheobronchial lumen; strength to oppose the oppression by expansive intra-extra parietal processes, blunt ends to prevent parietal injury caused by respiratory acts, provided with small protuberances on the outer wall to facilitate adhesion to the mucosa, preventing migration.

The stent created by Dumon marks the entrance into the modern era as far as the aim of ensuring the patency of the central airways is concerned; it will undergo several adjustments over the subsequent years, the largest one of which will be the use of self-expanding structure and / or materials in order to endorse adhesion, stability and efficiency of the prosthetic device, managed in the most appropriate way by interventional bronchoscopy. Regardless of the ongoing improvements there is still no ideal stent for the airways - and perhaps talking about it isn’t significant - because of the varied nature of the stenoses and their possible changes even during therapy.

The treatment of airway obstruction by stent essentially passes through three phases, each of which should be driven by clinicians. The first step is a thorough radiological assessment perfected by bronchoscopy, followed by the stent choice, and its placement and finally the follow up for detection and treatment of possible complications.

The advent of Multidetector Computed Tomography (MDCT) has dramatically transformed the non-invasive diagnostic imaging of the airways offering anatomical patterns i*n vivo;* interventional radiology procedures and, most of all, stent placement have experienced a great acceleration. Bronchoscopy remains the strategic procedure for the central airways, nevertheless MDCT imaging plays an essential role in the planning and guidance of bronchoscopic interventions and, most of all, providing a non invasive method for postprocedural surveillance. Volumetric isotropic acquisition ensures the detection of structures with a uniform geometry and high spatial resolution in all planes providing an extremely accurate anatomical documentation: it can be considered a problem solving tool. Paired inspiratory and dynamic expiratory MDCT imaging, along with newer cine CT imaging methods, have enhanced the assessment of tracheobronchomalacia in both adults and the pediatric population, increasing its diagnostic detection.

The systematic use of MDCT allows accurate preliminary depiction of the airways on three spatial planes with the aim of evaluating the nature of the stenosis to be treated, as well as its dimension, configuration and calibre. This preliminary study will be used by the interventional bronchoscopist for the most proper stent choice according to the clinical case. The goals to achieve (increasing the calibre of the airway, opposing the invasiveness of the expansive process, extended or localized, favouring the intraluminal debulking process of an invasive tumour, stabilizing of a condition of tracheobronchomalacia, sealing a tracheo-oesophageal fistula etc.) will be assessed in advance with careful evaluation of the overall pathological situation provided by MDCT, as well as with endoscopic observation. Although, on the basis of statistical results released so far, some Institutions are providing “recommendations” concerning the stent choice, it is the operator who must make the final choice, taking into account the specific situation of the patient. Moreover, even the imaging timing for re-evaluation of the stent condition, its relationships to the tracheobronchial wall and the possible complications, should be set by the bronchoscopist, preferably in agreement with the clinician and the radiologist. For example, the monitoring of a provisional stent (debulking of bulky tumors, treatment of transient or stabilized stenoses) must take into account the anatomical situation adjacent to the stenosis, even beyond the tracheobronchial tree.

Thus, MDCT has proven to be the most appropriate non invasive routine surveillance method in patients with airway stents [3], with a wider and longer vision compared to the endoscopic assessment of adverse events and complications: the continuous display of stents aids identification of stent complications, including migration, fracture, intraluminal granulation tissue and malignant proliferation.

The study by Ozgul et al. [4] describes the potential of MDCT through adequate clinical examples and two-dimensional imaging of the central airway stenting in helping to overcome the limitations of axial CT to visualize contemporarily the airways, the prosthesic stents by more accurately depicting the craniocaudal extent of airway abnormalities, improving the detection of subtle areas of stenosis, and helping to elucidate complex anatomic relationships.

I believe that the article’s most valuable quality is its contribution to familiarizing clinicians with this issue. The effectiveness of images, the procedures’ feasibility and the fidelity of the resulting images are very convincing, suggesting a development of clinical indications for treatments [5].

It is reasonable to assume that the gradual growth of confidence in the procedures and, above all, clinical-radiological-interventional specialities, will effectively contribute to improve patient selection and optimize decision-making measures in order to reduce complications and improve the quality of life of patients.
